# Resistance exercise, alone and in combination with aerobic exercise, and obesity in Dallas, Texas, US: A prospective cohort study

**DOI:** 10.1371/journal.pmed.1003687

**Published:** 2021-06-23

**Authors:** Angelique G. Brellenthin, Duck-chul Lee, Jason A. Bennie, Xuemei Sui, Steven N. Blair

**Affiliations:** 1 Department of Kinesiology, Iowa State University, Ames, Iowa, United States of America; 2 Centre for Health Research, University of Southern Queensland, Brisbane, Queensland, Australia; 3 Department of Exercise Science, University of South Carolina, Columbia, South Carolina, United States of America; Taipei Medical University, TAIWAN

## Abstract

**Background:**

Obesity is a significant and growing public health problem in high-income countries. Little is known about the relationship between resistance exercise (RE), alone and in combination with aerobic exercise (AE), and the risk of developing obesity. The purpose of this prospective cohort study was to examine the associations between different amounts and frequencies of RE, independent of AE, and incident obesity.

**Methods and findings:**

Participants were 11,938 healthy adults ages 18–89 years with a BMI < 30 kg/m^2^ at baseline who completed at least 2 clinical examinations during 1987–2005 as part of the Aerobics Center Longitudinal Study. Self-reported RE participation in minutes/week and days/week was collected from a standardized questionnaire. Incident obesity was defined as a BMI ≥ 30 kg/m^2^ at follow-up. Incident obesity was also defined by waist circumference (WC) > 102/88 cm for men/women and percent body fat (PBF) ≥ 25%/30% for men/women at follow-up in participants who were not obese by WC (*n =* 9,490) or PBF (*n =* 8,733) at baseline. During the average 6-year follow-up, 874 (7%), 726 (8%), and 1,683 (19%) developed obesity defined by BMI, WC, or PBF, respectively. Compared with no RE, 60–119 min/wk of RE was associated with 30%, 41%, and 31% reduced risk of obesity defined by BMI (hazard ratio [95% CI], 0.70 [0.54–0.92], *p* = 0.008), WC (0.59 [0.44–0.81], *p <* 0.001), and PBF (0.69 [0.57–0.83], *p <* 0.001), respectively, after adjusting for confounders including age, sex, examination year, smoking status, heavy alcohol consumption, hypertension, hypercholesterolemia, diabetes, and AE. Compared with not meeting the RE guidelines of ≥2 d/wk, meeting the RE guidelines was associated with 18%, 30%, and 30% reduced risk of obesity defined by BMI (hazard ratio [95% CI], 0.82 [0.69–0.97], *p* = 0.02), WC (0.70 [0.57–0.85], *p <* 0.001), and PBF (0.70 [0.62–0.79], *p <* 0.001), respectively. Compared with meeting neither guideline, meeting both the AE and RE guidelines was associated with the smallest hazard ratios for obesity. Limitations of this study include limited generalizability as participants were predominantly white men from middle to upper socioeconomic strata, use of self-reported RE, and lack of detailed diet data for the majority of participants.

**Conclusions:**

In this study, we observed that RE was associated with a significantly reduced risk of obesity even after considering AE. However, meeting both the RE and AE guidelines was associated with the lowest risk of obesity.

## Abstract 2

In a prospective cohort study, Angelique G. Brellenthin and colleagues investigate associations between resistance and aerobic exercise, and incident obesity in the United States.

## Author summary

**Why was this study done?:**

- Although many people know that aerobic exercises like brisk walking or jogging can help prevent obesity, it is not clear whether resistance exercises can also help prevent obesity.
- This study was conducted to determine the relationship between resistance exercise and the risk of developing obesity, even after accounting for participation in aerobic exercise.

**What did the researchers do and find?:**

- We identified 11,938 adults without obesity who reported their participation in resistance and aerobic exercises as well as several other demographic and lifestyle variables.
- Participants were followed for an average of 6 years to determine who did and did not develop obesity as determined by body mass index, waist circumference, and percent body fat.
- Compared to individuals who did not perform resistance exercise (71% of participants), those who performed 1–2 hours/week or at least 2 days/week of resistance exercise had a 20%–30% reduced risk of obesity, even after adjusting for aerobic exercise.
- Resistance exercise was associated with a reduced risk of obesity defined by body mass index, waist circumference, and percent body fat, although the strongest relationships between resistance exercise and obesity were consistently observed for obesity defined by percent body fat.

**What do these findings mean?:**

- Resistance exercise may reduce the risk of obesity, especially obesity characterized by higher body fat percentage.
- These results suggest that resistance exercise should be promoted alongside aerobic exercise as part of comprehensive physical activity programs to prevent obesity.

## Introduction

The prevalence of obesity in the United States (US) is projected to grow from 40% to 50% by 2030 [1,2]. This increase in obesity over the next decade would contribute to an excess of 8 million diabetes diagnoses, 7 million cardiovascular disease diagnoses, and 0.5 million cancer diagnoses [2]. Obesity is typically treated through lifestyle modifications, such as diet and exercise, that induce a caloric deficit. Modest weight loss (approximately 5% body weight) is associated with significantly improved cardiovascular and metabolic health [3,4]. However, more than 50% of individuals do not sustain a ≥5% weight loss for more than 5 years, highlighting the considerable challenges of treating obesity in the long term [5]. Considering the rising obesity rates, the comorbid and costly diseases that often follow obesity, and the difficulty of sustained weight loss, identifying effective strategies that prevent obesity in the first place is critical.

There is strong evidence that physical activity (PA), particularly aerobic exercise (AE; e.g., running), helps prevent weight gain [6]. However, the relationship between resistance exercise (RE), a major component of the US physical activity guidelines [7], and obesity is not clear. In general, RE is associated with a lower prevalence of obesity from cross-sectional studies [8–10]. RE is also often included as part of weight-loss trials to preserve metabolically active lean (muscle) tissue [11,12]. Thus, the available evidence suggests that RE may also be an effective obesity prevention strategy, although there are limited data.

Body mass index (BMI) ≥ 30 kg/m^2^ is the most commonly used metric to define obesity [13,14], but BMI has a clear limitation in that it provides no information on body composition and adipose distribution. Investigating other adiposity measures, such as waist circumference (WC) or percent body fat (PBF), may have valuable clinical implications and allow a more comprehensive understanding of the relationship between RE and incident obesity. Therefore, we investigated 3 adiposity measures (BMI, WC, and PBF) in this study. The purpose of this study was to examine the long-term associations between RE, independent of and combined with AE, and incident obesity in a large cohort. We hypothesized that RE would be independently associated with a reduced risk of developing obesity, but that combined AE and RE would be associated with the lowest risk of obesity.

## Methods

### Study participants

This study is reported as per the Strengthening the Reporting of Observational Studies in Epidemiology (STROBE) guideline (S1 STROBE Checklist). Participants were 15,094 men and women 18–89 years old from the prospective Aerobics Center Longitudinal Study (ACLS) who had complete data from at least 2 clinical examinations at the Cooper Clinic in Dallas, Texas, during the period 1 January 1987 to 31 December 2005. Each clinical examination included anthropometric and body composition measurements. Participants were excluded if they had a history of heart attack, stroke, or cancer that would significantly affect RE participation and/or body weight at baseline (*n =* 1,194) or if they had a baseline BMI ≥ 30 kg/m^2^ (*n =* 1,962). For the WC- and PBF-specific analyses, we further excluded participants if they had obesity defined by WC (*n =* 2,448) and PBF (*n =* 3,205), respectively, at baseline. The final samples included 11,938 in the BMI, 9,490 in the WC, and 8,733 in the PBF analyses.

Demographic characteristics (i.e., age and sex), lifestyle behaviors (i.e., smoking, alcohol consumption, and PA), and personal medical history (i.e., hypertension, hypercholesterolemia, and diabetes) were determined using a standardized medical history questionnaire. Participants were categorized as either current smokers or never/former smokers. Heavy alcohol drinking was defined as >14 and >7 drinks/week for men and women, respectively [15]. Responses to the aerobic PA questions (e.g., frequency, duration) were transformed into metabolic equivalent (MET)–minutes/week, and participants were categorized into 4 AE levels based on whether they accumulated 0, 1–499, 500–999, or ≥1,000 MET-min/wk. Participants were also categorized as meeting the 2018 PA guidelines for AE if they accumulated ≥500 MET-min/wk of activity [7]. Hypertension, hypercholesterolemia, and diabetes are common chronic conditions that may have a bidirectional relationship with weight gain [16,17]. Presence of hypertension, hypercholesterolemia, and diabetes was identified through self-report of physician diagnosis or by measured values of blood pressure ≥ 130/80 mm Hg [18], total cholesterol ≥ 13.3 mmol/L [19], and fasting glucose ≥ 7.0 mmol/L [20], respectively. The Cooper Clinic Institute institutional review board provided oversight of the study, and written informed consent was obtained from participants at each time point. Additional details regarding the ACLS study sample and methods have been described previously [21–23].

### Assessment of resistance exercise

Participants were asked if they were “currently involved in a muscle strengthening program,” and if so, they reported their average frequency (days/week) and duration (minutes) of RE at baseline. Exposure categories were based on the total amount (0, 1–59, 60–119, 120–179, or ≥180 min/wk) or frequency (0, 1, 2, 3, 4, or ≥5 d/wk) of RE. Meeting the RE guidelines was defined as ≥2 d/wk [7].

### Assessment of obesity

The primary outcome was obesity defined by BMI, WC, or PBF. BMI was calculated by dividing body weight (kilograms) by height (meters) squared. A BMI ≥ 30 kg/m^2^ was considered obese for both men and women [13,14]. WC (centimeters) was assessed by trained researchers using an inelastic tape measure at the level of the umbilicus. Abdominal obesity was defined as having a WC > 102 cm for men or >88 cm for women [24]. PBF was determined through either hydrostatic weighing or skinfold measurements following standardized protocols previously described [25]. When both were available, PBF derived from hydrostatic weighing was used over skinfold estimates of body fat. Given there are no widely accepted risk categories or cut points for PBF, we followed the methods of previous studies in this cohort and considered ≥25% for men and ≥30% for women as PBF-defined obesity [23,26].

### Statistical analyses

The ACLS was designed to examine the associations between various health indices and behaviors, specifically cardiorespiratory fitness and PA, and numerous health outcomes over time. All analyses were prespecified, but the study did not have a written analysis plan. Descriptive statistics of the sample were calculated by categories of weekly RE time. Cox proportional hazards regression was performed to compute hazard ratios (HRs) and 95% confidence intervals (CIs) for incident obesity defined by BMI, WC, or PBF across weekly RE times and frequencies. Follow-up time was calculated between baseline and the earliest follow-up exam when participants developed obesity (cases) or their last follow-up exam (non-cases). Cox regression was also used to test for linear trends across exposure groups by including RE exposure categories as continuous rather than categorical variables. Regression models were adjusted for age, sex, and baseline examination year in model 1; additionally for smoking status, heavy alcohol drinking, hypertension, hypercholesterolemia, and diabetes in model 2; and then additionally for AE in model 3. Covariates were selected based on their inclusion in prior observational studies of PA and obesity as well as their recognized associations with PA and obesity [8,26–28].

Stratified Cox regression was also used to test the potential effect modification of RE on each obesity outcome by subgroups based on sex, age (<50 years or ≥50 years), current smoking, heavy alcohol drinking, presence of at least 1 chronic medical condition (i.e, hypertension, hypercholesterolemia, or diabetes), follow-up time (<5 years or ≥5 years), and meeting the AE guidelines. Participants were also dichotomized into groups based on whether they met neither, either, or both the AE (≥500 MET-min/wk) and RE (≥2 d/wk) guidelines [7] to examine the joint associations of each combination of meeting the guidelines with incident obesity.

Finally, we conducted sensitivity analyses. In the first sensitivity analysis, we considered another common alternative cut point of ≥35% body fat for women in PBF analyses [29,30]. In the second sensitivity analysis, we investigated the potential effects of baseline body weight as well as baseline BMI, WC, and PBF by additionally adjusting for body weight (kilograms, continuous) in the final model or through stratified analysis, i.e., analysis of the association between RE and incident obesity stratified by categories of BMI, WC, and PBF. For the BMI-stratified analysis, participants were categorized as having normal weight (<25 kg/m^2^) or overweight (25–29.9 kg/m^2^) at baseline. For the WC-stratified analyses, strata were based on the risk categories for metabolic disorders identified by the International Diabetes Federation, where <80 cm for women or <94 cm for men is considered low risk, and 80–88 cm for women and 94–102 cm for men is considered moderate risk [31]. Since there are no well-established cut points for body fat percentages and health outcomes, a sex-specific median split of baseline PBF was used to define the lower and upper PBF strata. In the third sensitivity analysis, we investigated the effect of total energy intake (kilocalories) as a covariate in a subsample of 4,002 participants (34%) who had complete 3-day diet records available.

We tested the proportional hazards assumption by examining log–log survival plots grouped on the exposures, and no violations were found. All analyses were 2-sided with a significance level of *p <* 0.05. Analyses were conducted using SAS version 9.4 (SAS Institute, Cary, NC, US).

## Results

The baseline characteristics of the sample by weekly RE time are shown in Table 1. On average, participants were 47 years old with a normal-weight BMI (mean = 24.8, SD = 2.7 kg/m^2^) at baseline. Most participants (71%) did not participate in any RE. After an average follow-up of 5.6 years (SD = 4.5; interquartile range 2–8), 874 (7%) of the 11,938 participants developed obesity defined by BMI. In the WC subsample, 726 (8%) of the 9,490 participants developed obesity defined by WC during an average follow-up of 5.6 years (SD = 4.5; interquartile range 2–8). In the PBF subsample, 1,683 (19%) of the 8,733 participants developed obesity defined by PBF during an average follow-up of 5.5 years (SD = 4.4; interquartile range 2–8). S1 Table depicts the baseline characteristics by the analytical samples (i.e., entire sample, WC subsample, and PBF subsample). As there was overlap in participants who developed obesity by 1 or more definitions, S2 Table lists the obesity case types and combinations by baseline adiposity status.

**Table 1:** Baseline characteristics of the 11,938 participants by weekly minutes of resistance exercise.

Characteristic	Resistance exercise (minutes per week)					*p*-Value
	0 (*n =* 8,504)	1–59 (*n =* 824)	60–119 (*n =* 1297)	120–179 (*n =* 622)	≥180 (*n =* 691)	
Age (y), mean (SD)	47.3 (10.0)	46.0 (8.4)	46.1 (9.1)	44.9 (9.5)	43.7 (10.1)	<0.001
Sex (female), *n* (%)	1,925 (23)	144 (17)	308 (24)	171 (27)	156 (23)	<0.001
Body mass index (kg/m^2^), mean (SD)	24.9 (2.7)	24.7 (2.6)	24.5 (2.7)	24.5 (2.8)	24.6 (2.7)	<0.001
Waist circumference (cm), mean (SD)	88.0 (16.4)	86.8 (10.1)	85.6 (10.3)	84.3 (11.0)	85.1 (10.1)	<0.001
Percent body fat, mean (SD)*	22.3 (5.9)	20.3 (5.5)	20.2 (6.2)	20.1 (6.2)	18.6 (6.0)	<0.001
Current smoking, *n* (%)	976 (11)	69 (8)	117 (9)	70 (11)	63 (9)	0.004
Heavy alcohol drinking, *n* (%)^†^	1,046 (12)	93 (11)	169 (13)	70 (11)	79 (11)	0.65
Systolic blood pressure (mm Hg), mean (SD)	119.6 (14.1)	119.4 (13.2)	118.7 (13.5)	118.4 (12.8)	119.8 (13.9)	0.06
Diastolic blood pressure (mm Hg), mean (SD)	79.7 (9.7)	79.9 (9.2)	79.1 (9.1)	79.2 (9.5)	79.8 (9.4)	0.13
Hypertension, *n* (%)^‡^	5,033 (59)	497 (60)	744 (57)	362 (58)	420 (61)	0.53
Total cholesterol (mmol/L), mean (SD)	11.6 (2.4)	11.1 (2.0)	11.0 (2.1)	10.9 (1.9)	10.7 (2.0)	<0.001
Hypercholesterolemia, *n* (%)^§^	2,660 (31)	225 (27)	323 (25)	147 (24)	145 (21)	<0.001
Fasting glucose (mmol/L), mean (SD)	5.4 (1.0)	5.4 (0.7)	5.4 (0.7)	5.4 (0.6)	5.4 (0.7)	0.002
Diabetes, *n* (%)^ǁ^	276 (3)	30 (4)	36 (3)	18 (3)	14 (2)	0.34
Total aerobic physical activity (MET-min/wk), mean (SD)	925.5 (1,235.5)	1,538.3 (1,358.0)	1,742.7 (2,091.3)	1,650.5 (1,462.9)	1,852.5 (1,756.9)	<0.001
Aerobic activity level, *n* (%)						
0 MET-min/wk	2,717 (32)	40 (5)	56 (4)	34 (6)	48 (7)	<0.001
1–499 MET-min/wk	1,292 (15)	112 (14)	143 (11)	83 (13)	86 (12)	<0.001
500–999 MET-min/wk	1,488 (18)	173 (21)	297 (23)	130 (21)	123 (18)	<0.001
≥1,000 MET-min/wk	3,007 (35)	499 (61)	801 (62)	375 (60)	434 (63)	<0.001
Resistance exercise (min/wk), mean (SD)	0 (0)	33.9 (11.5)	77.7 (15.8)	130.5 (13.0)	248.4 (115.3)	<0.001

*Fifty-seven percent of participants had hydrostatic weighing-derived percent body fat, and 43% of participants had skinfold-derived percent body fat.

^†^Defined as >14 drinks/week for men and >7 drinks/week for women.

^‡^Self-report of physician diagnosis or measured blood pressure ≥ 130/80 mm Hg.

^§^Self-report of physician diagnosis or measured total cholesterol ≥ 13.3 mmol/L.

^ǁ^Self-report of physician diagnosis or measured fasting glucose ≥ 7.0 mmol/L.

As shown in Table 2, performing any RE (mean = 111 min/wk; median = 90 min/wk) was associated with a 19%, 31%, and 32% reduced risk of developing obesity defined by BMI, WC, and PBF, respectively, compared with no RE (0 min/wk), after adjusting for potential confounders including AE (model 3). The strongest linear dose–response relationship between weekly RE time and obesity was observed for PBF (*p* for linear trend < 0.001).

**Table 2:** Hazard ratios for incident obesity as defined by body mass index, waist circumference, or percent body fat by weekly minutes of resistance exercise.

Obesity outcome	Resistance exercise amount	Number (%) of participants	Number of cases	Hazard ratio (95% CI) or *p*-value for linear trend			
				Unadjusted model	Model 1*	Model 2^†^	Model 3^‡^
Body mass index ≥ 30 kg/m^2^^§^ (*n =* 11,938)	**Minutes per week**						
	0	8,504 (71)	666	1.00 [reference]	1.00 [reference]	1.00 [reference]	1.00 [reference]
	1–59	824 (7)	56	0.89 (0.68–1.17)	0.76 (0.58–1.00)	0.78 (0.59–1.03)	0.86 (0.65–1.13)
	60–119	1,297 (11)	65	**0.75 (0.58–0.97)**	**0.61 (0.47–0.79)**	**0.64 (0.49–0.83)**	**0.70 (0.54–0.92)**
	120–179	622 (5)	44	1.18 (0.87–1.60)	0.88 (0.65–1.20)	0.90 (0.66–1.23)	0.99 (0.73–1.36)
	≥180	691 (6)	43	0.91 (0.67–1.24)	**0.72 (0.53–0.98)**	**0.73 (0.54–0.996)**	0.81 (0.59–1.10)
				*p =* 0.35	***p <* 0.001**	***p =* 0.002**	***p =* 0.05**
	**Any resistance exercise**						
	No (0 min/wk)	8,504 (71)	666	1.00 [reference]	1.00 [reference]	1.00 [reference]	1.00 [reference]
	Yes (≥1 min/wk)	3,434 (29)	208	0.89 (0.76–1.04)	**0.72 (0.61–0.84)**	**0.74 (0.63–0.87)**	**0.81 (0.69–0.96)**
Waist circumference: >102 cm for men, >88 for women^ǁ^ (*n =* 9,490)	**Minutes per week**						
	0	6,618 (70)	582	1.00 [reference]	1.00 [reference]	1.00 [reference]	1.00 [reference]
	1–59	694 (7)	44	**0.73 (0.54–0.99)**	**0.69 (0.51–0.94)**	**0.71 (0.52–0.96)**	0.78 (0.57–1.06)
	60–119	1,076 (11)	45	**0.55 (0.41–0.74)**	**0.52 (0.38–0.70)**	**0.54 (0.39–0.73)**	**0.59 (0.44–0.81)**
	120–179	517 (6)	28	0.79 (0.53–1.14)	0.70 (0.48–1.03)	0.72 (0.49–1.06)	0.80 (0.54–1.18)
	≥180	585 (6)	27	**0.59 (0.40–0.87)**	**0.57 (0.38–0.83)**	**0.57 (0.39–0.84)**	**0.64 (0.43–0.94)**
				***p <* 0.001**	***p <* 0.001**	***p <* 0.001**	***p <* 0.001**
	**Any resistance exercise**						
	No (0 min/wk)	6,618 (70)	582	1.00 [reference]	1.00 [reference]	1.00 [reference]	1.00 [reference]
	Yes (≥1 min/wk)	2,872 (30)	144	**0.64 (0.54–0.77)**	**0.61 (0.50–0.73)**	**0.62 (0.51–0.75)**	**0.69 (0.57–0.83)**
Percent body fat: ≥25% for men, ≥30% for women^¶^ (*n =* 8,733)	**Minutes per week**						
	0	5,980 (68)	1,323	1.00 [reference]	1.00 [reference]	1.00 [reference]	1.00 [reference]
	1–59	650 (7)	105	**0.73 (0.60–0.89)**	**0.71 (0.58–0.86)**	**0.72 (0.59–0.88)**	**0.76 (0.62–0.93)**
	60–119	1,020 (12)	131	**0.64 (0.54–0.77)**	**0.63 (0.52–0.75)**	**0.64 (0.54–0.77)**	**0.69 (0.57–0.83)**
	120–179	499 (6)	65	**0.71 (0.56–0.91)**	**0.67 (0.52–0.86)**	**0.68 (0.53–0.88)**	**0.73 (0.56–0.94)**
	≥180	584 (7)	59	**0.49 (0.37–0.63)**	**0.49 (0.38–0.64)**	**0.50 (0.39–0.65)**	**0.53 (0.41–0.70)**
				***p <* 0.001**	***p <* 0.001**	***p <* 0.001**	***p <* 0.001**
	**Any resistance exercise**						
	No (0 min/wk)	5,980 (68)	1,323	1.00 [reference]	1.00 [reference]	1.00 [reference]	1.00 [reference]
	Yes (≥1 min/wk)	2,753 (32)	360	**0.64 (0.57–0.72)**	**0.63 (0.56–0.71)**	**0.64 (0.57–0.72)**	**0.68 (0.60–0.77)**

*Adjusted for age, sex, and examination year.

^†^Adjusted for model 1 plus current smoking, heavy alcohol drinking, hypertension, hypercholesterolemia, and diabetes.

^‡^Adjusted for model 2 plus aerobic physical activity category (0, 1–499, 500–999, or ≥1,000 MET-min/wk).

^§^All participants had a baseline body mass index < 30 kg/m^2^.

^ǁ^Excludes 2,448 participants with baseline waist circumference > 102/88 cm for men/women.

^¶^Excludes 3,205 participants with baseline percent body fat ≥ 25%/30% for men/women.

Bolded values indicate significant hazard ratios (95% CI).

As shown in Table 3, meeting the RE guidelines of ≥2 d/wk was associated with a 18%, 30%, and 30% reduced risk of developing obesity defined by BMI, WC, and PBF, respectively, compared with not meeting the RE guidelines (<2 d/wk), after adjusting for potential confounders including AE (model 3). The strongest linear dose–response relationship between weekly RE frequency and obesity was observed for PBF (*p* for linear trend < 0.001).

**Table 3:** Hazard ratios for incident obesity as defined by body mass index, waist circumference, or percent body fat by weekly frequency of resistance exercise.

Obesity outcome	Resistance exercise frequency	Number (%) of participants	Number of cases	Hazard ratio (95% CI) or *p*-value for linear trend			
				Unadjusted model	Model 1*	Model 2^†^	Model 3^‡^
Body mass index ≥ 30 kg/m^2^^§^ (*n =* 11,938)	**Days per week**						
	0	8,504 (71)	666	1.00 [reference]	1.00 [reference]	1.00 [reference]	1.00 [reference]
	1	253 (2)	14	1.02 (0.60–1.73)	0.72 (0.42–1.23)	0.74 (0.73–1.25)	0.80 (0.47–1.35)
	2	927 (8)	52	0.88 (0.66–1.16)	**0.69 (0.52–0.92)**	**0.72 (0.54–0.95)**	0.79 (0.59–1.05)
	3	1,509 (13)	107	1.00 (0.81–1.22)	0.83 (0.67–1.01)	0.85 (0.69–1.04)	0.94 (0.76–1.16)
	4	413 (3)	18	**0.58 (0.37–0.93)**	**0.48 (0.30–0.76)**	**0.49 (0.31–0.79)**	**0.55 (0.34–0.88)**
	≥5	332 (3)	17	0.73 (0.45–1.18)	**0.61 (0.38–0.99)**	0.62 (0.38–1.01)	0.69 (0.42–1.11)
				*p =* 0.06	***p <* 0.001**	***p <* 0.001**	***p =* 0.01**
	**Meeting resistance exercise guidelines**						
	No (<2 d/wk)	8,757 (73)	680	1.00 [reference]	1.00 [reference]	1.00 [reference]	1.00 [reference]
	Yes (≥2 d/wk)	3,181 (27)	194	0.88 (0.75–1.03)	**0.72 (0.62–0.85)**	**0.75 (0.63–0.88)**	**0.82 (0.69–0.97)**
Waist circumference: >102 cm for men, >88 for women^ǁ^ (*n =* 9,490)	**Days per week**						
	0	6,618 (70)	582	1.00 [reference]	1.00 [reference]	1.00 [reference]	1.00 [reference]
	1	218 (2)	9	0.67 (0.35–1.30)	0.61 (0.31–1.17)	0.62 (0.32–1.20)	0.67 (0.35–1.30)
	2	773 (8)	31	**0.55 (0.38–0.79)**	**0.52 (0.36–0.75)**	**0.54 (0.37–0.78)**	**0.60 (0.41–0.87)**
	3	1,260 (13)	79	**0.77 (0.61–0.98)**	**0.73 (0.58–0.93)**	**0.75 (0.59–0.95)**	0.83 (0.65–1.06)
	4	352 (4)	13	**0.43 (0.25–0.74)**	**0.40 (0.23–0.70)**	**0.41 (0.24–0.72)**	**0.46 (0.27–0.80)**
	≥5	269 (3)	12	**0.55 (0.31–0.98)**	**0.51 (0.29–0.90)**	**0.50 (0.28–0.90)**	**0.56 (0.31–0.99)**
				***p <* 0.001**	***p <* 0.001**	***p <* 0.001**	***p <* 0.001**
	**Meeting resistance exercise guidelines**						
	No (<2 d/wk)	6,836 (72)	591	1.00 [reference]	1.00 [reference]	1.00 [reference]	1.00 [reference]
	Yes (≥2 d/wk)	2,654 (28)	135	**0.65 (0.54–0.78)**	**0.61 (0.51–0.74)**	**0.63 (0.52–0.76)**	**0.70 (0.57–0.85)**
Percent body fat: ≥25% for men, ≥30% for women^¶^ (*n =* 8,733)	**Days per week**						
	0	5,980 (68)	1,323	1.00 [reference]	1.00 [reference]	1.00 [reference]	1.00 [reference]
	1	198 (2)	21	**0.61 (0.40–0.94)**	**0.58 (0.38–0.90)**	**0.59 (0.38–0.91)**	**0.62 (0.40–0.95)**
	2	745 (9)	89	**0.62 (0.50–0.77)**	**0.62 (0.50–0.77)**	**0.63 (0.51–0.79)**	**0.68 (0.54–0.84)**
	3	1,203 (14)	181	**0.73 (0.62–0.85)**	**0.71 (0.61–0.83)**	**0.73 (0.62–0.85)**	**0.78 (0.66–0.91)**
	4	337 (4)	37	**0.48 (0.34–0.66)**	**0.47 (0.34–0.65)**	**0.48 (0.34–0.66)**	**0.51 (0.37–0.71)**
	≥5	270 (3)	32	**0.57 (0.40–0.81)**	**0.53 (0.37–0.75)**	**0.54 (0.38–0.77)**	**0.58 (0.41–0.83)**
				***p <* 0.001**	***p <* 0.001**	***p <* 0.001**	***p <* 0.001**
	**Meeting resistance exercise guidelines**						
	No (<2 d/wk)	6,178 (71)	1,344	1.00 [reference]	1.00 [reference]	1.00 [reference]	1.00 [reference]
	Yes (≥2 d/wk)	2,555 (29)	339	**0.65 (0.58–0.73)**	**0.64 (0.57–0.72)**	**0.65 (0.58–0.74)**	**0.70 (0.62–0.79)**

*Adjusted for age, sex, and examination year.

^†^Adjusted for model 1 plus current smoking, heavy alcohol drinking, hypertension, hypercholesterolemia, and diabetes.

^‡^Adjusted for model 2 plus aerobic physical activity category (0, 1–499, 500–999, or ≥1,000 MET-min/wk).

^§^All participants had a baseline body mass index < 30 kg/m^2^.

^ǁ^Excludes 2,448 participants with baseline waist circumference > 102/88 cm for men/women.

^¶^Excludes 3,205 participants with baseline percent body fat ≥ 25%/30% for men/women.

Bolded values indicate significant hazard ratios (95% CI).

**Fig 1:**
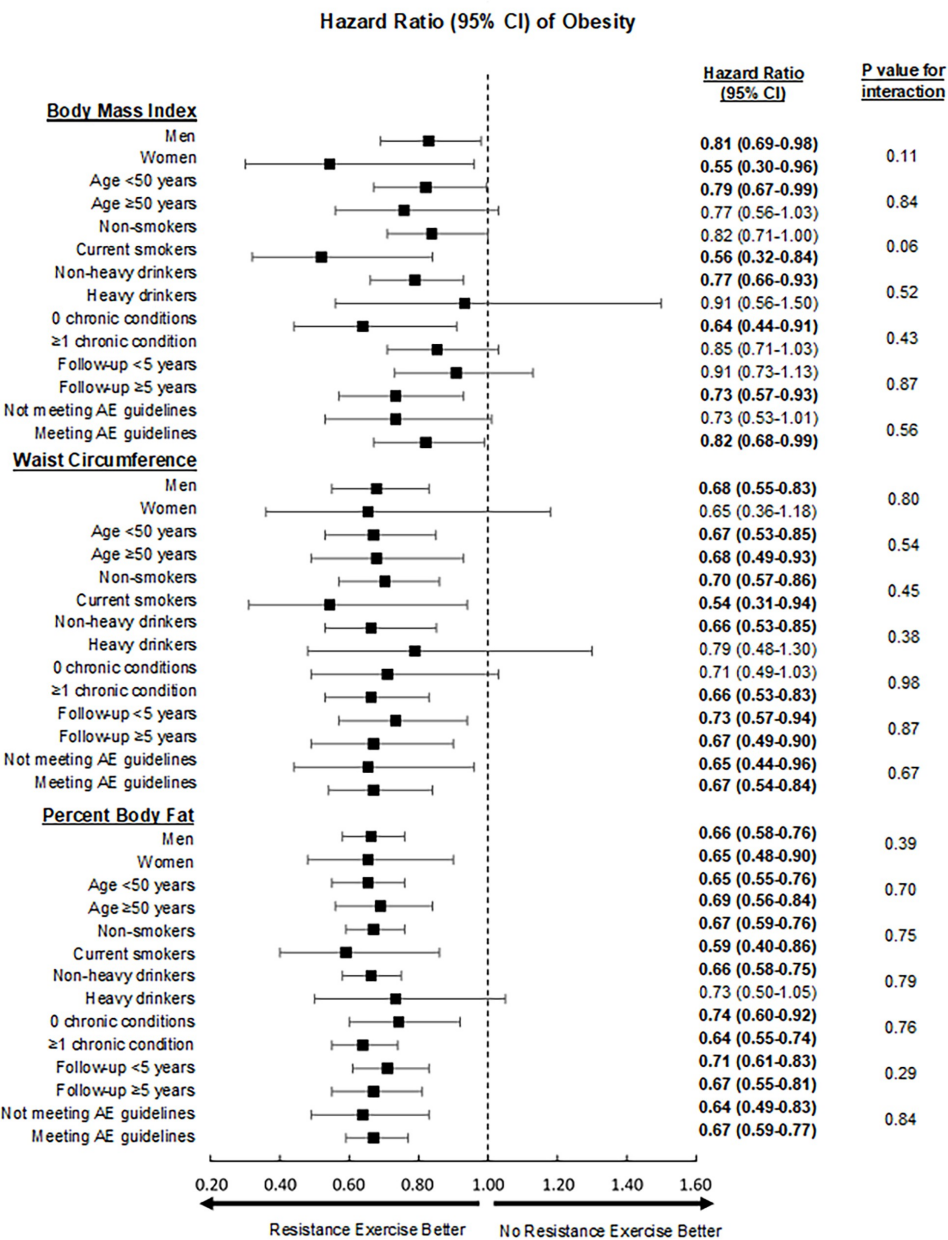
Hazard ratios of obesity defined by body mass index, waist circumference, and percent body fat in stratified subgroup analyses. Hazard ratios are depicted by black boxes, and 95% confidence intervals by whiskers. The reference group for all analyses was those who performed 0 min/wk of resistance exercise. The model was adjusted for examination year, age (not in age-stratified analysis), sex (not in sex-stratified analysis), current smoking (not in smoking-stratified analysis), heavy alcohol drinking (not in drinking-stratified analysis), presence of ≥1 chronic condition (not in chronic-condition-stratified analysis), and meeting aerobic exercise (AE) guidelines (not in AE-guideline-stratified analysis).

Stratified analyses are illustrated in Fig 1. We found no significant interactions (all *p-*values > 0.05), and HRs of obesity by participation in RE versus no RE were similar and favoring RE (all HRs below 1.00) for all subgroups.

**Fig 2:**
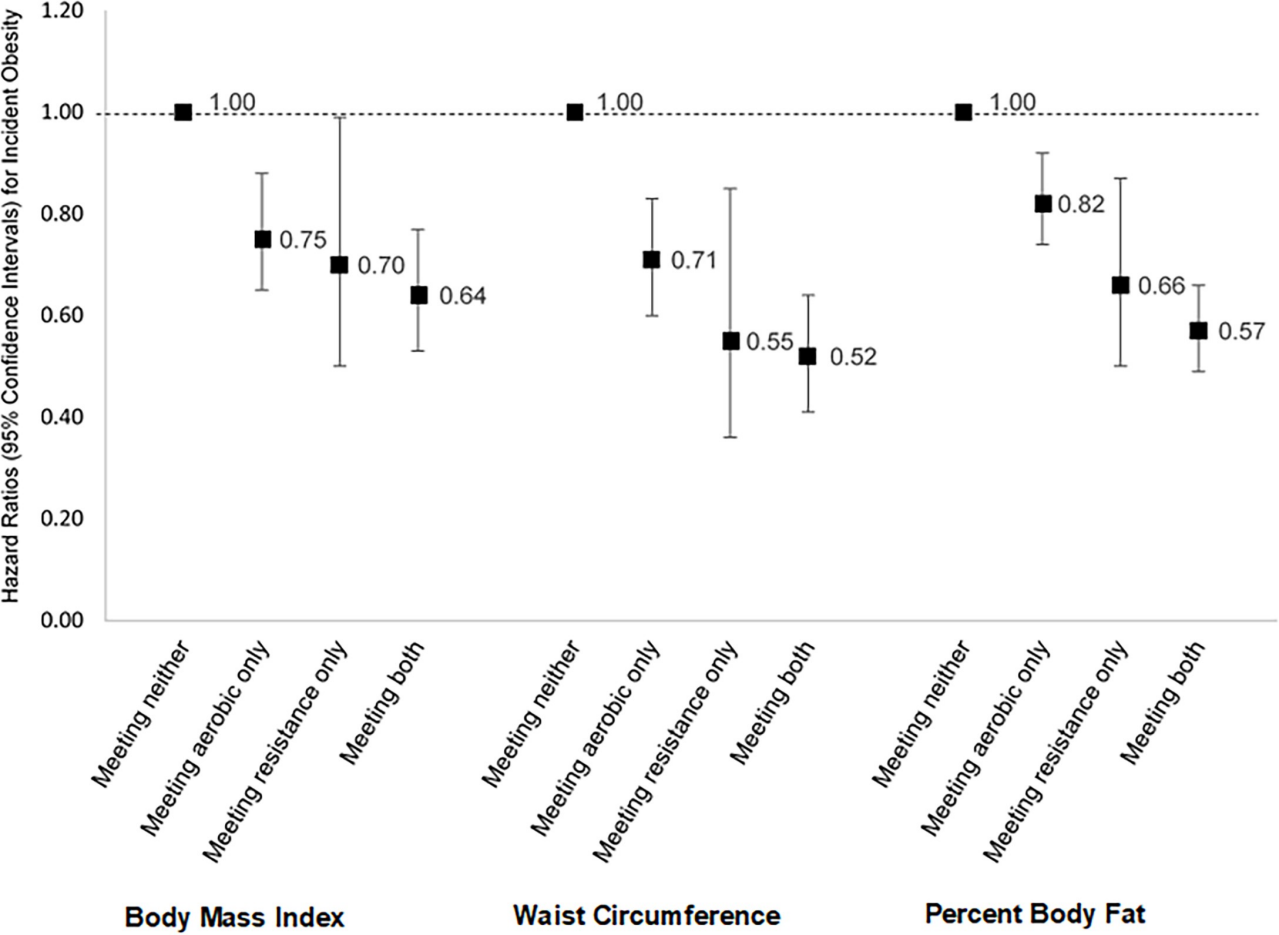
Hazard ratios for incident obesity by whether participants met the aerobic and/or resistance exercise guidelines. Hazard ratios are depicted by black boxes, and 95% confidence intervals by whiskers. Participants were categorized into 4 groups based on whether they met neither guideline, aerobic exercise guidelines only, resistance exercise guidelines only, or both aerobic and resistance exercise guidelines. The group of participants who met neither guideline served as the reference. The model was adjusted for age, sex, examination year, current smoking, and heavy alcohol drinking. The cases/participants for the body mass index analysis were 357/4,075, 323/4,682, 36/536, and 158/2,645 for the neither, aerobic only, resistance only, and both groups, respectively. The cases/participants for the waist circumference analysis were 301/3,067, 290/3,769, 23/450, and 112/2,204 for the neither, aerobic only, resistance only, and both groups, respectively. The cases/participants for the percent body fat analysis were 588/2,577, 756/3,601, 55/397, and 284/2,158 for the neither, aerobic only, resistance only, and both groups, respectively.

As shown in Fig 2, compared to meeting neither guideline, the lowest HRs of obesity were among those meeting both guidelines, followed by RE guidelines, and then AE guidelines. An additional joint analysis using those who met only the AE guidelines as the reference indicated that meeting both guidelines was associated with a significantly lower risk of obesity defined by WC (HR [95% CI], 0.73 [0.59–0.91]) and PBF (0.69 [0.60–0.80]), but not BMI (0.84 [0.70–1.02]).

We found similar results in the sensitivity analyses when we used the alternate PBF cut point of ≥35% in women. After additionally adjusting for baseline body weight in the final model, the association between RE and obesity defined by BMI was no longer significant (*p* > 0.05); however, the associations of RE with WC- and PBF-defined obesity outcomes remained significant. Stratified analyses based on subgroups of baseline BMI and WC indicated there were no significant interactions, with non-significant risk reductions ranging from 10% to 17% for obesity among the groups performing RE compared with no RE. The lower and upper 50th percentiles of baseline PBF were associated with significantly reduced risks of 24% and 18% of developing obesity, respectively, among the group who performed RE compared with no RE (S3 Table). In the subsample of 4,002 participants with total energy intake data, all HRs remained below 1.00, but the BMI and WC results became non-significant in all 3 models, before and after adjustment for total energy intake, possibly due to the smaller sample size. However, even with the smaller sample size, RE was associated with a significantly reduced risk of obesity defined by PBF in the final model (HR [95% CI], 0.76 [0.63–0.93]) after further adjusting for total energy intake.

## Discussion

The primary finding from this study is that participating in RE was associated with 20%–30% reduced risk of developing obesity defined by BMI, WC, or PBF. A 20%–30% risk reduction is comparable to the 20%–30% reduced risk of cardiovascular disease observed when adults from the general population with average cholesterol levels are prescribed short-term statin therapy [32]. The associations between RE and obesity were consistent among various subgroups (e.g., men/women, older/younger participants) and persisted after adjusting for several potential confounders, including AE. Compared with no RE, performing RE for 1 to 2 h/wk (mean = 78 min/wk; median = 80 min/wk) was associated with the lowest risk of developing obesity, suggesting that additional amounts of RE might not be necessary to help prevent obesity. Meeting the current RE guidelines of ≥2 d/wk was also associated with a similar obesity risk reduction (20%–30%). In the joint analysis, meeting either AE or RE guidelines was associated with a significantly lower risk of obesity compared with meeting neither guideline, but meeting both guidelines was associated with the lowest risk of incident obesity.

A unique aspect of this study was the use of multiple adiposity measures, which revealed consistent, but somewhat nuanced, associations between RE and obesity. For example, high amounts of RE (e.g., ≥3 h/wk or ≥5 d/wk) were not associated with a reduced risk of obesity defined by BMI. However, these same high amounts of RE were significantly associated with a reduced risk of obesity defined by WC or PBF. Thus, it is possible that individuals regularly performing high amounts of RE and possibly increasing their muscle mass may be classified as obese according to BMI, which does not take into account fat distribution and body composition, as WC and PBF do. The dose–response associations between RE and obesity were the strongest for PBF, further highlighting the protective potential of RE against “excessive fat accumulation,” which is the physiological definition of obesity that may be more strongly associated with adverse clinical outcomes [13,27,33,34]. There is preclinical evidence supporting the notion that regular RE may increase the likelihood that a modest caloric surplus (e.g., consuming calories in excess of the body’s caloric requirements) will support lean mass growth (e.g., muscle hypertrophy) preferentially over fat tissue accumulation [35,36]. The robust associations between RE and obesity defined by PBF even in the various sensitivity analyses may also represent the important role that RE has in maintaining a favorable body composition, including maintaining/increasing lean mass and minimizing fat gain with age [37]. Maintenance of lean mass is particularly critical to prevent sarcopenia and maintain quality of life and independence in later life [38], which could also promote greater PA participation and better long-term weight management.

Since there are limited prospective observational studies on RE and incident obesity, we are unable to directly compare our findings with other studies. However, in our earlier cross-sectional study in a nationally representative sample of nearly 400,000 US adults, we found that more frequent RE was associated with a lower prevalence of obesity [39]. Likewise, a cross-sectional study in a national sample of 1.7 million US adults found that meeting either the RE or AE guidelines was associated with lower prevalence of obesity, and the lowest prevalence of obesity was observed among those who met both guidelines, which is similar to our findings [8]. Another cross-sectional study found that the prevalence of obesity was significantly lower among women who met both guidelines compared with those who met only the AE guidelines [9], which aligns with our joint analysis results suggesting that those who met both guidelines had a lower risk of obesity when those who met only the AE guidelines were the reference. Our findings are also consistent with a prospective study in over 10,000 men that found AE and RE were inversely associated with WC changes over 12 years, although incident obesity was not assessed [28]. Finally, in our earlier study in men, we found that muscular strength, a fitness parameter often used to reflect recent participation in RE, was linearly associated with a reduced risk of WC- and PBF-defined obesity [26].

There are several potential mechanisms through which RE might help prevent obesity. RE may increase basal metabolic rate, which constitutes the largest portion of daily caloric expenditure [40,41]. This is because RE helps preserve or increase metabolically active lean mass (e.g., muscle) that typically decreases with age [37]. Performing RE also requires increased caloric expenditure, although not typically to the same magnitude as an equivalent duration of AE [42]. However, RE increases basal metabolic rate for up to 24 hours after an exercise session [43,44]. The prolonged effects of RE on basal metabolic rate may partially explain why those who met only the RE guidelines and those who met both the AE and RE guidelines had relatively lower risks of obesity than the those who met only the AE guidelines. Furthermore, RE may stimulate muscle hypertrophy, which has been found to cause reductions in fat mass, potentially due to increases in glycolysis and fat oxidation throughout the body [45,46]. This may explain why the associations between RE and obesity were weaker using BMI as the outcome, which takes into account only crude body mass, including muscle.

### Limitations

This study has the following limitations. First, generalizability is limited as participants were primarily white, non-Hispanic, and well-educated individuals of middle to upper socioeconomic standing, so the results cannot be generalized to more diverse populations [47]. However, the homogeneity of the sample minimizes the potential effects of differences in race or ethnicity, education, and income on the results, which are factors associated with obesity [1,48,49]. Furthermore, the prevalence of RE participation in this sample is similar to that in other nationally representative data [50,51]. Second, the results are likely influenced by unmeasured confounding factors such as mental health or time spent in sedentary behaviors; of particular note is the lack of dietary data for the entire sample, since diet is a strong contributor to weight gain and obesity. In the subsample of 4,002 participants with diet data, adding total energy intake as a covariate did not change the associations between RE and obesity, although many results, even in the basic model, were not significant, possibly due to the smaller sample size. Also, 11,439 participants (96%) had available data on “number of meals per week,” which was significantly correlated with total energy intake (*p* < 0.001) in the dietary data subsample. When including meals per week as a proxy for total energy intake in the final model with the larger sample size, the associations between RE and obesity outcomes remained either significant or not significant as they were in the primary analyses, suggesting that the potential confounding effects of diet-related factors on the associations were similar across the RE groups. Research generally shows that food consumption patterns are fairly stable within individuals although there have been population-level patterns of increased caloric consumption over time that would be similar across all participants in all groups [52,53]. Nonetheless, more active individuals tend to have healthier diets [54,55], so our results should be interpreted with caution. To provide more robust estimates of the associations between RE and obesity in the future, large observational studies may want to consider using a more rigorous dietary data collection method, such as the Automated Self-Administered 24-Hour (ASA24) Dietary Assessment Tool developed and supported by the National Institutes of Health [56]. Third, this study used self-reported PA as the exposure, which is typically overestimated compared to objective measures [57]. Thus, it is possible that the associations between RE and obesity in this study could be underestimated compared to results derived using objective measures of RE, although objective methods to assess RE participation at the population level are extremely limited at this time. Furthermore, our measure of RE did not capture perceived intensity of RE, which may also affect the observed associations. Other demographic and lifestyle variables such as smoking and alcohol consumption were also self-reported and could be prone to recall or social-desirability bias. Finally, this was an observational study, indicating that causal relationships between RE and obesity cannot be inferred.

A major strength of this study is its prospective design, which allowed us to examine the long-term association of RE with incident obesity in a large population, which has not been previously reported. The ACLS has data on objectively measured BMI, WC, and PBF in both men and women, which expands upon many previous studies that used solely BMI. However, it should be noted that WC measured at the umbilicus in this study may underestimate the true waist circumference compared to other measurements (e.g., top of iliac crest). Our large sample also has detailed data on both weekly total amount and frequency of AE and RE, making it possible to compare the relative importance for the risk of obesity of meeting the AE and RE components of the 2018 PA guidelines.

## Conclusions

Obesity in high-income countries including the US has been increasing at an alarming rate over several decades. We found that RE was independently associated with reduced risk of developing obesity using 3 adiposity measures (BMI, WC, and PBF). Specifically, meeting the 2018 RE guidelines of ≥2 d/wk or 1–2 h/wk of RE was associated with a reduced risk of developing obesity regardless of whether individuals met the AE guidelines. While it is still not clear if RE alone is sufficient to reduce the risk of developing obesity at the population level, these results clearly suggest that performing both RE and AE may be the best approach to prevent obesity. However, 71% of participants reported no engagement in RE. This might be due to the fact that in contrast to AE, RE has rarely been a focus of PA promotion [58]. There may also be additional perceived or actual barriers that RE requires a gym membership, expensive equipment, or more instruction or education to perform it safely, and indeed, our previous study showed that those with a gym membership were more likely to meet the RE guidelines [59]. However, effective and practical RE can be performed with minimal equipment (e.g., resistance bands) or body weight exercises with simple guidance that is freely available through a variety of sources (e.g., online instructional videos). Future studies as well as health promotion strategies should consider these potential barriers in order to determine the relative effectiveness and accessibility of various RE programs (e.g., body weight exercises and weight machines) to improve health, manage weight, and prevent chronic disease.

## Supporting information

S1 STROBE Checklist Strengthening the Reporting of Observational Studies in Epidemiology (STROBE) statement checklist. (DOCX)

S1 Table Baseline characteristics by analytical sample. (DOCX)

S2 Table Obesity case types and combinations by baseline adiposity status. (DOCX)

S3 Table Hazard ratios for incident obesity by participation in resistance exercise stratified by baseline body mass index, waist circumference, and percent body fat. (DOCX)

## Abbreviations

ACLS: Aerobics Center Longitudinal Study
AE: aerobic exercise
MET: metabolic equivalent
PA: physical activity
PBF: percent body fat
RE: resistance exercise
WC: waist circumference
