# Supplementary material for: Resistance exercise, alone and in combination with aerobic exercise, and obesity in Dallas, Texas, US: A prospective cohort study
Source: PLoS Med. 2021 Jun 23;18(6):e1003687. doi: 10.1371/journal.pmed.1003687 (PMC8266085; doi:10.1371/journal.pmed.1003687)
Supplement: S1 Table — (DOCX) [file pmed.1003687.s002.docx]

| **S1 Table. Baseline characteristics by analytical sample** | | | |
| --- | --- | --- | --- |
| **Characteristic** | **BMI (entire) sample (N=11,938)** | **WC sample (n=9,490)** | **PBF sample (n=8,733)** |
| Age, mean (SD), y | 46.7 (9.8) | 46.6 (9.8) | 45.9 (9.9) |
| Sex (female), No. (%) | 2,704 (22.7) | 1,680 (17.7) | 1,907 (21.8) |
| Body mass index, mean (SD), kg/m^2^ | 24.8 (2.7) | 24.8 (2.5) | 24.3 (2.6) |
| Waist circumference, mean (SD), cm | 87.3 (15.0) | 86.9 (9.8) | 85.6 (12.8) |
| Percent body fat, mean (SD), % | 21.6 (6.0) | 20.9 (5.8) | 19.5 (4.7) |
| Total aerobic physical activity, mean (SD), MET-min/wk | 1,148.0 (1,450.9) | 1,180.7 (1,444.2) | 1,280.1 (1,533.4) |
| Resistance exercise, mean (SD), min/wk | 32.0 (70.6) | 33.6 (71.8) | 35.8 (75.1) |
| BMI=body mass index; WC= waist circumference; PBF=percent body fat | | | |
