# Supplementary material for: Resistance exercise, alone and in combination with aerobic exercise, and obesity in Dallas, Texas, US: A prospective cohort study
Source: PLoS Med. 2021 Jun 23;18(6):e1003687. doi: 10.1371/journal.pmed.1003687 (PMC8266085; doi:10.1371/journal.pmed.1003687)
Supplement: S2 Table — (DOCX) [file pmed.1003687.s003.docx]

| **S2 Table. Obesity case types and combinations by baseline adiposity status** | | | | |
| --- | --- | --- | --- | --- |
|  | **Total sample not obese by BMI=11,938** | | | |
|  | **Not obese by BMI, WC, and PBF**  **7,779 (65%)** | **Not obese by BMI only**  **1,494 (12%)** | **Not obese by WC and BMI only**  **1,711 (14%)** | **Not obese by PBF and BMI only**  **954 (8%)** |
| **Total BMI cases=874** | | | | |
| BMI only case | 119 | 60 | 26 | 12 |
| BMI and WC case | 27 | 6 | 4 | 17 |
| BMI and PBF case | 74 | 17 | 53 | 10 |
| BMI, WC, and PBF case | 154 | 140 | 102 | 53 |
| **Total WC cases=726** | | | | |
| WC only case | 103 | -- | 16 | -- |
| WC and BMI case | 27 | -- | 4 | -- |
| WC and PBF case | 189 | -- | 131 | -- |
| WC, BMI, and PBF case | 154 | -- | 102 | -- |
| **Total PBF cases=1,683** | | | | |
| PBF only case | 999 | -- | -- | 142 |
| PBF and BMI case | 74 | -- | -- | 10 |
| PBF and WC case | 189 | -- | -- | 62 |
| PBF, BMI, and WC case | 154 | -- | -- | 53 |
| BMI=body mass index; WC= waist circumference; PBF=percent body fat. -- indicates that the participants were not included in the WC or PBF case count or analyses due to having obesity by WC or PBF, respectively, at baseline. | | | | |
