# Supplementary material for: Resistance exercise, alone and in combination with aerobic exercise, and obesity in Dallas, Texas, US: A prospective cohort study
Source: PLoS Med. 2021 Jun 23;18(6):e1003687. doi: 10.1371/journal.pmed.1003687 (PMC8266085; doi:10.1371/journal.pmed.1003687)
Supplement: S3 Table — (DOCX) [file pmed.1003687.s004.docx]

**S3 Table. Hazard ratios for incident obesity by participation in resistance exercise stratified by baseline body mass index, waist circumference, and percent body fat**

| **Obesity Outcome** | **Resistance Exercise** | **No. of Participants** | **No. of Cases** | **Hazard Ratio (95% CI)*** | **P-value for interaction** |
| --- | --- | --- | --- | --- | --- |
| **Body Mass Index** |  |  |  |  |  |
| <25 kg/m^2^ |  |  |  |  | 0.33 |
|  | 0 min/wk | 4,163 | 49 | 1.00 [reference] |  |
|  | ≥1 min/wk | 1,889 | 14 | 0.83 (0.44-1.57) |  |
| ≥25 kg/m^2^ |  |  |  |  |  |
|  | 0 min/wk | 4,341 | 617 | 1.00 [reference] |  |
|  | ≥1 min/wk | 1,545 | 194 | 0.90 (0.76-1.07) |  |
| **Waist Circumference** |  |  |  |  |  |
| ≤80 cm for women;  ≤94 cm for men |  |  |  |  | 0.80 |
|  | 0 min/wk | 4,375 | 104 | 1.00 [reference] |  |
|  | ≥1 min/wk | 2,251 | 41 | 0.88 (0.60-1.29) |  |
| 80-88 cm for women;  94-102 cm for men |  |  |  |  |  |
|  | 0 min/wk | 2,243 | 478 | 1.00 [reference] |  |
|  | ≥1 min/wk | 621 | 103 | 0.87 (0.70-1.10) |  |
| **Percent Body Fat^†^** |  |  |  |  |  |
| ≤23.5% for women;  ≤19.2% for men |  |  |  |  | 0.40 |
|  | 0 min/wk | 2,696 | 265 | 1.00 [reference] |  |
|  | ≥1 min/wk | 1,664 | 99 | **0.76 (0.59-0.96)** |  |
| >23.5% for women;  >19.2% for men |  |  |  |  |  |
|  | 0 min/wk | 3,284 | 1058 | 1.00 [reference] |  |
|  | ≥1 min/wk | 1,089 | 261 | **0.82 (0.71-0.94)** |  |

*Adjusted for age, sex, examination year, smoking, heavy alcohol drinking, hypertension, hypercholesterolemia, diabetes and aerobic physical activity category (0, 1-499, 500-999, or ≥1,000 MET-min/wk).

^†^Percent body fat groups defined based on a median split of body fat values for men and women at baseline.
